# Bis(2-amino-4,5-dimethyl­anilinium chloride) 4,5-dimethyl­benz­ene-1,2-diamine monohydrate

**DOI:** 10.1107/S1600536809013816

**Published:** 2009-04-18

**Authors:** Seik Weng Ng

**Affiliations:** a Department of Chemistry, University of Malaya, 50603 Kuala Lumpur, Malaysia

## Abstract

The title compound, 2C_8_H_13_N_2_^+^·2Cl^−^·C_8_H_12_N_2_·H_2_O, is a hydrated 2:1 cocrystal of the 2-amino-4,5-dimethyl­anilinium chloride salt and the 4,5-dimethyl­benz­ene-1,2-diamine free base. An intra­molecular N—H⋯N hydrogen bond occurs in one of the organic mol­ecules. In the crystal structure, the components are linked by N—H⋯Cl, N—H⋯N, N—H⋯O and O—H⋯Cl hydrogen bonds into a layered motif.

## Related literature

4,5-Dimethyl­phenyl­ene-1,2-diamine is used in the synthesis of benzimidazoles; see: El Ashry *et al.* (1986 ▶). The crystal structures of several metal complexes of 4,5-dimethyl­phenyl­ene-1,2-diamine have been reported; see: Pérez-Cabré *et al.* (2004 ▶); Eremenko *et al.* (2005 ▶); Kiskin *et al.* (2006 ▶); Malkov *et al.* (2003 ▶); Mikhailova *et al.* (2002 ▶); Redshaw *et al.* (1992 ▶).
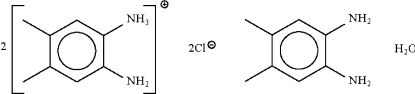


## Experimental

### 

#### Crystal data

2C_8_H_13_N_2_^+^·2Cl^−^·C_8_H_12_N_2_·H_2_O
*M* *_r_* = 499.52
Monoclinic, 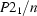

*a* = 11.7102 (5) Å
*b* = 6.0938 (3) Å
*c* = 35.948 (1) Å
β = 91.257 (2)°
*V* = 2564.7 (2) Å^3^
*Z* = 4
Mo *K*α radiation
μ = 0.28 mm^−1^
*T* = 123 K
0.40 × 0.12 × 0.02 mm

#### Data collection

Bruker SMART APEX diffractometer
Absorption correction: multi-scan (*SADABS*; Sheldrick, 1996 ▶) *T*_min_ = 0.896, *T*_max_ = 0.994
16985 measured reflections
5877 independent reflections
3608 reflections with *I* > 2σ(*I*)
*R*_int_ = 0.087

#### Refinement

*R*[*F* ^2^ > 2σ(*F* ^2^)] = 0.061
*wR*(*F* ^2^) = 0.166
*S* = 1.06
5877 reflections
368 parameters
27 restraints
H atoms treated by a mixture of independent and constrained refinement
Δρ_max_ = 0.47 e Å^−3^
Δρ_min_ = −0.45 e Å^−3^

### 

Data collection: *APEX2* (Bruker, 2008 ▶); cell refinement: *SAINT* (Bruker, 2008 ▶); data reduction: *SAINT*; program(s) used to solve structure: *SHELXS97* (Sheldrick, 2008 ▶); program(s) used to refine structure: *SHELXL97* (Sheldrick, 2008 ▶); molecular graphics: *X-SEED* (Barbour, 2001 ▶); software used to prepare material for publication: *publCIF* (Westrip, 2009 ▶).

## Supplementary Material

Crystal structure: contains datablocks global, I. DOI: 10.1107/S1600536809013816/hb2941sup1.cif

Structure factors: contains datablocks I. DOI: 10.1107/S1600536809013816/hb2941Isup2.hkl

Additional supplementary materials:  crystallographic information; 3D view; checkCIF report

## Figures and Tables

**Table 1:** Hydrogen-bond geometry (Å, °)

*D*—H⋯*A*	*D*—H	H⋯*A*	*D*⋯*A*	*D*—H⋯*A*
O1—H1⋯Cl1	0.85 (2)	2.37 (2)	3.212 (3)	172 (4)
O1—H2⋯Cl1^i^	0.85 (4)	2.82 (3)	3.402 (3)	128 (4)
O1—H2⋯Cl1^ii^	0.85 (4)	2.73 (4)	3.331 (2)	129 (3)
N1—H12⋯Cl1^i^	0.88 (3)	2.81 (3)	3.661 (3)	164 (3)
N2—H21⋯Cl1	0.87 (2)	2.61 (3)	3.361 (3)	145 (3)
N2—H22⋯N1	0.87 (4)	2.49 (4)	2.810 (4)	102 (3)
N2—H22⋯Cl1^ii^	0.87 (4)	2.79 (4)	3.599 (3)	155 (3)
N3—H31⋯N2	0.89 (2)	2.08 (2)	2.927 (4)	160 (2)
N3—H32⋯Cl1^i^	0.89 (3)	2.22 (3)	3.092 (3)	168 (2)
N3—H33⋯Cl2	0.89 (2)	2.347 (19)	3.216 (3)	167 (2)
N4—H41⋯Cl2^i^	0.87 (2)	2.38 (3)	3.233 (3)	165 (3)
N4—H42⋯Cl1^i^	0.87 (3)	2.63 (3)	3.434 (3)	155 (3)
N5—H51⋯O1	0.88 (2)	1.88 (2)	2.758 (3)	172 (3)
N5—H52⋯Cl2^iii^	0.89 (2)	2.354 (19)	3.242 (3)	176 (2)
N5—H53⋯Cl2	0.89 (2)	2.75 (3)	3.176 (3)	111 (2)
N5—H53⋯Cl2^iv^	0.89 (2)	2.730 (19)	3.540 (3)	153 (2)
N6—H61⋯Cl2^iv^	0.88 (3)	2.71 (3)	3.408 (3)	138 (2)
N6—H62⋯N1^ii^	0.88 (2)	2.45 (3)	3.277 (5)	156 (3)

Symmetry codes: (i) 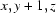; (ii) 
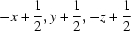
; (iii) 
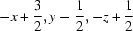
; (iv) 
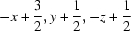
.

**Fig. 1.**
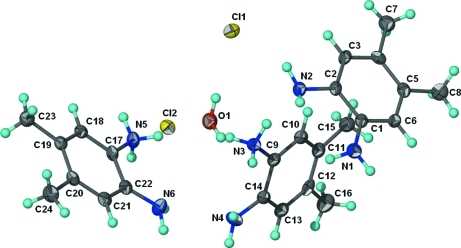
The molecular structure of (I) showing 70% displacement ellipsoids Hydrogen atoms are drawn as spheres of arbitrary radius.

## supplementary crystallographic information

### Experimental

Colourless plates of (I) were unexpectedly isolated from the reaction of dibenzyltin dichloride (1 mmol) and 4,5-dimethylphenene-1,2-diamine in ethanol, in an attempt at synthesizing a tin complex.  Atmospheric water was presumably incorporated into the crystal.

### Refinement

The carbon-bound H-atoms were placed in calculated positions (C—H 0.95 to 0.98 Å) and refined as riding with *U*_iso_(H) = 1.2*U*_eq_(C) or 1.5U_eq_(methyl C).

The amino/ammonium and water H-atoms were located in a difference map, and were refined with distance restraint of N–H = 0.88 + 0.01 Å and H···H = 1.44±0.01; O–H = 0.84±0.01 Å and H···H = 1.37±0.01 Å; their U_iso_ values were freely refined.

### Crystal data

**Table:**

2C_8_H_13_N_2_^+^·2Cl^−^·C_8_H_12_N_2_·H_2_O	*F*(000) = 1072
*M**_r_* = 499.52	*D*_x_ = 1.294 Mg m^−^^3^
Monoclinic, *P*2_1_/*n*	Mo *K*α radiation, λ = 0.71073 Å
Hall symbol: -P 2yn	Cell parameters from 1367 reflections
*a* = 11.7102 (5) Å	θ = 2.3–21.3°
*b* = 6.0938 (3) Å	µ = 0.28 mm^−^^1^
*c* = 35.948 (1) Å	*T* = 123 K
β = 91.257 (2)°	Plate, colourless
*V* = 2564.7 (2) Å^3^	0.40 × 0.12 × 0.02 mm
*Z* = 4	

### Data collection

**Table:**

Bruker SMART APEX diffractometer	5877 independent reflections
Radiation source: fine-focus sealed tube	3608 reflections with *I* > 2σ(*I*)
graphite	*R*_int_ = 0.087
ω scans	θ_max_ = 27.5°, θ_min_ = 1.1°
Absorption correction: multi-scan (*SADABS*; Sheldrick, 1996)	*h* = −15→15
*T*_min_ = 0.896, *T*_max_ = 0.994	*k* = −7→7
16985 measured reflections	*l* = −46→46

### Refinement

**Table:**

Refinement on *F*^2^	Primary atom site location: structure-invariant direct methods
Least-squares matrix: full	Secondary atom site location: difference Fourier map
*R*[*F*^2^ > 2σ(*F*^2^)] = 0.061	Hydrogen site location: inferred from neighbouring sites
*wR*(*F*^2^) = 0.166	H atoms treated by a mixture of independent and constrained refinement
*S* = 1.06	*w* = 1/[σ^2^(*F*_o_^2^) + (0.0711*P*)^2^] where *P* = (*F*_o_^2^ + 2*F*_c_^2^)/3
5877 reflections	(Δ/σ)_max_ = 0.001
368 parameters	Δρ_max_ = 0.47 e Å^−^^3^
27 restraints	Δρ_min_ = −0.45 e Å^−^^3^

### Special details

**Table:**

Geometry. All e.s.d.'s (except the e.s.d. in the dihedral angle between two l.s. planes) are estimated using the full covariance matrix. The cell e.s.d.'s are taken into account individually in the estimation of e.s.d.'s in distances, angles and torsion angles; correlations between e.s.d.'s in cell parameters are only used when they are defined by crystal symmetry. An approximate (isotropic) treatment of cell e.s.d.'s is used for estimating e.s.d.'s involving l.s. planes.

### Fractional atomic coordinates and isotropic or equivalent isotropic displacement parameters (Å^2^)

**Table:**

	*x*	*y*	*z*	*U*_iso_*/*U*_eq_	
Cl1	0.34435 (7)	−0.08992 (14)	0.23602 (2)	0.0211 (2)	
Cl2	0.73644 (7)	0.46400 (14)	0.21359 (2)	0.0195 (2)	
O1	0.4381 (2)	0.3932 (4)	0.25526 (7)	0.0223 (5)	
H1	0.407 (3)	0.269 (3)	0.2515 (12)	0.056 (16)*	
H2	0.386 (3)	0.480 (5)	0.2618 (15)	0.09 (2)*	
N1	0.1622 (3)	0.7021 (5)	0.15983 (8)	0.0221 (7)	
H11	0.134 (3)	0.828 (3)	0.1521 (10)	0.045 (13)*	
H12	0.206 (3)	0.723 (6)	0.1798 (8)	0.063 (16)*	
N2	0.2902 (2)	0.3227 (5)	0.17615 (8)	0.0187 (6)	
H21	0.309 (3)	0.188 (3)	0.1818 (10)	0.044 (13)*	
H22	0.247 (4)	0.380 (6)	0.1931 (10)	0.077 (18)*	
N3	0.4870 (2)	0.6160 (5)	0.18456 (8)	0.0182 (6)	
H31	0.439 (2)	0.508 (4)	0.1786 (9)	0.047 (13)*	
H32	0.454 (2)	0.696 (5)	0.2021 (7)	0.055 (15)*	
H33	0.5496 (15)	0.557 (4)	0.1946 (8)	0.020 (9)*	
N4	0.5951 (3)	1.0243 (5)	0.19219 (8)	0.0226 (7)	
H41	0.623 (3)	1.156 (3)	0.1953 (9)	0.033 (11)*	
H42	0.547 (3)	0.991 (5)	0.2094 (7)	0.034 (11)*	
N5	0.6403 (2)	0.4368 (5)	0.29539 (7)	0.0192 (6)	
H51	0.5748 (14)	0.436 (5)	0.2828 (8)	0.039 (12)*	
H52	0.675 (2)	0.309 (3)	0.2918 (8)	0.017 (9)*	
H53	0.684 (2)	0.543 (3)	0.2867 (10)	0.049 (14)*	
N6	0.5083 (3)	0.7947 (5)	0.31996 (8)	0.0225 (7)	
H61	0.549 (3)	0.828 (5)	0.3006 (7)	0.039 (12)*	
H62	0.480 (3)	0.914 (3)	0.3300 (9)	0.037 (12)*	
C1	0.1979 (3)	0.5659 (5)	0.13072 (8)	0.0163 (7)	
C2	0.2533 (3)	0.3666 (5)	0.13923 (8)	0.0164 (7)	
C3	0.2794 (3)	0.2259 (6)	0.11040 (9)	0.0176 (7)	
H3	0.3158	0.0903	0.1161	0.021*	
C4	0.2541 (3)	0.2765 (6)	0.07322 (9)	0.0181 (7)	
C5	0.2002 (3)	0.4753 (6)	0.06478 (9)	0.0184 (7)	
C6	0.1728 (3)	0.6154 (6)	0.09380 (9)	0.0182 (7)	
H6	0.1356	0.7501	0.0881	0.022*	
C7	0.2850 (3)	0.1172 (6)	0.04304 (9)	0.0240 (8)	
H7A	0.3161	−0.0171	0.0543	0.036*	
H7B	0.3423	0.1837	0.0271	0.036*	
H7C	0.2166	0.0814	0.0281	0.036*	
C8	0.1731 (3)	0.5393 (6)	0.02498 (9)	0.0238 (8)	
H8A	0.1289	0.6758	0.0246	0.036*	
H8B	0.1285	0.4225	0.0128	0.036*	
H8C	0.2444	0.5610	0.0117	0.036*	
C9	0.5147 (3)	0.7433 (5)	0.15144 (9)	0.0165 (7)	
C10	0.4943 (3)	0.6604 (6)	0.11619 (9)	0.0178 (7)	
H10	0.4577	0.5221	0.1135	0.021*	
C11	0.5260 (3)	0.7745 (6)	0.08461 (9)	0.0189 (7)	
C12	0.5794 (3)	0.9785 (6)	0.08938 (9)	0.0197 (7)	
C13	0.5985 (3)	1.0614 (6)	0.12482 (9)	0.0207 (7)	
H13	0.6342	1.2006	0.1275	0.025*	
C14	0.5672 (3)	0.9480 (6)	0.15672 (9)	0.0185 (7)	
C15	0.5057 (3)	0.6771 (6)	0.04653 (9)	0.0214 (8)	
H15A	0.4568	0.5473	0.0485	0.032*	
H15B	0.5789	0.6349	0.0360	0.032*	
H15C	0.4681	0.7859	0.0303	0.032*	
C16	0.6182 (3)	1.1097 (6)	0.05619 (10)	0.0284 (9)	
H16A	0.6576	1.2428	0.0649	0.043*	
H16B	0.5517	1.1506	0.0407	0.043*	
H16C	0.6705	1.0208	0.0415	0.043*	
C17	0.6155 (3)	0.4633 (6)	0.33487 (8)	0.0166 (7)	
C18	0.6513 (3)	0.3053 (6)	0.35998 (9)	0.0164 (7)	
H18	0.6924	0.1814	0.3514	0.020*	
C19	0.6281 (3)	0.3241 (6)	0.39766 (9)	0.0176 (7)	
C20	0.5686 (3)	0.5114 (6)	0.40946 (8)	0.0169 (7)	
C21	0.5321 (3)	0.6663 (6)	0.38371 (9)	0.0183 (7)	
H21A	0.4912	0.7907	0.3922	0.022*	
C22	0.5531 (3)	0.6466 (5)	0.34573 (9)	0.0170 (7)	
C23	0.6664 (3)	0.1477 (6)	0.42444 (9)	0.0226 (8)	
H23A	0.7183	0.2110	0.4433	0.034*	
H23B	0.7060	0.0321	0.4109	0.034*	
H23C	0.5997	0.0853	0.4366	0.034*	
C24	0.5461 (3)	0.5479 (6)	0.45022 (9)	0.0231 (8)	
H24A	0.4901	0.6661	0.4529	0.035*	
H24B	0.6175	0.5886	0.4632	0.035*	
H24C	0.5161	0.4126	0.4611	0.035*	

### Atomic displacement parameters (Å^2^)

**Table:**

	*U*^11^	*U*^22^	*U*^33^	*U*^12^	*U*^13^	*U*^23^
Cl1	0.0203 (4)	0.0185 (4)	0.0246 (4)	0.0007 (3)	0.0029 (3)	0.0014 (3)
Cl2	0.0167 (4)	0.0210 (4)	0.0209 (4)	−0.0039 (3)	0.0022 (3)	0.0002 (3)
O1	0.0174 (12)	0.0246 (14)	0.0250 (13)	−0.0005 (12)	0.0000 (10)	0.0010 (11)
N1	0.0231 (16)	0.0224 (17)	0.0207 (16)	0.0022 (14)	−0.0007 (13)	−0.0035 (13)
N2	0.0186 (15)	0.0185 (16)	0.0189 (15)	−0.0029 (13)	−0.0023 (12)	0.0046 (12)
N3	0.0171 (14)	0.0151 (15)	0.0223 (15)	−0.0023 (13)	−0.0008 (12)	0.0013 (12)
N4	0.0234 (16)	0.0199 (17)	0.0244 (16)	−0.0070 (13)	0.0001 (13)	−0.0029 (13)
N5	0.0158 (14)	0.0259 (18)	0.0160 (14)	0.0036 (13)	−0.0005 (12)	−0.0020 (12)
N6	0.0245 (16)	0.0235 (17)	0.0196 (16)	0.0050 (14)	0.0012 (13)	0.0049 (13)
C1	0.0139 (15)	0.0179 (18)	0.0172 (16)	−0.0006 (14)	0.0002 (12)	0.0009 (14)
C2	0.0139 (16)	0.0182 (18)	0.0169 (16)	−0.0025 (13)	−0.0012 (12)	0.0040 (13)
C3	0.0126 (16)	0.0172 (18)	0.0229 (17)	0.0018 (13)	−0.0003 (13)	0.0033 (14)
C4	0.0165 (16)	0.0181 (18)	0.0197 (17)	−0.0043 (14)	0.0017 (13)	−0.0037 (14)
C5	0.0156 (16)	0.0206 (18)	0.0188 (16)	−0.0032 (14)	−0.0012 (13)	0.0009 (14)
C6	0.0148 (16)	0.0159 (18)	0.0236 (17)	−0.0010 (14)	−0.0034 (13)	0.0019 (14)
C7	0.0222 (18)	0.024 (2)	0.0257 (18)	0.0004 (16)	0.0005 (15)	−0.0042 (15)
C8	0.0251 (18)	0.026 (2)	0.0203 (17)	0.0016 (16)	0.0028 (14)	0.0003 (15)
C9	0.0132 (16)	0.0161 (17)	0.0203 (17)	0.0007 (13)	0.0010 (13)	0.0025 (13)
C10	0.0101 (15)	0.0176 (18)	0.0257 (18)	0.0018 (13)	0.0007 (13)	−0.0021 (14)
C11	0.0137 (16)	0.0206 (19)	0.0222 (17)	0.0020 (14)	−0.0004 (13)	0.0008 (14)
C12	0.0138 (16)	0.0219 (19)	0.0233 (17)	0.0058 (14)	0.0007 (13)	0.0066 (14)
C13	0.0148 (16)	0.0168 (18)	0.0305 (19)	0.0019 (14)	−0.0011 (14)	0.0033 (15)
C14	0.0120 (15)	0.0168 (18)	0.0267 (17)	0.0019 (14)	−0.0008 (13)	−0.0025 (14)
C15	0.0212 (18)	0.0211 (19)	0.0219 (18)	0.0018 (15)	0.0012 (14)	−0.0011 (14)
C16	0.028 (2)	0.028 (2)	0.030 (2)	−0.0002 (17)	0.0019 (16)	0.0093 (16)
C17	0.0121 (15)	0.0220 (18)	0.0156 (15)	0.0002 (14)	−0.0005 (12)	−0.0016 (14)
C18	0.0126 (16)	0.0174 (18)	0.0193 (16)	−0.0002 (13)	0.0004 (13)	−0.0028 (14)
C19	0.0133 (16)	0.0175 (17)	0.0219 (17)	−0.0045 (14)	−0.0023 (13)	0.0019 (14)
C20	0.0125 (15)	0.0212 (18)	0.0171 (16)	−0.0028 (14)	0.0000 (12)	−0.0022 (14)
C21	0.0095 (15)	0.0218 (19)	0.0236 (17)	−0.0007 (14)	0.0005 (13)	−0.0037 (14)
C22	0.0115 (15)	0.0197 (18)	0.0198 (17)	−0.0034 (14)	0.0004 (13)	0.0004 (14)
C23	0.0237 (18)	0.022 (2)	0.0220 (18)	−0.0005 (15)	−0.0026 (14)	0.0032 (14)
C24	0.0235 (18)	0.028 (2)	0.0178 (17)	0.0007 (16)	0.0019 (14)	−0.0021 (15)

### Geometric parameters (Å, °)

**Table:**

O1—H1	0.85 (1)	C8—H8A	0.9800
O1—H2	0.85 (4)	C8—H8B	0.9800
N1—C1	1.406 (4)	C8—H8C	0.9800
N1—H11	0.88 (1)	C9—C10	1.380 (4)
N1—H12	0.87 (4)	C9—C14	1.401 (5)
N2—C2	1.412 (4)	C10—C11	1.389 (4)
N2—H21	0.87 (1)	C10—H10	0.9500
N2—H22	0.87 (4)	C11—C12	1.400 (5)
N3—C9	1.463 (4)	C11—C15	1.506 (4)
N3—H31	0.89 (3)	C12—C13	1.384 (5)
N3—H32	0.89 (3)	C12—C16	1.514 (5)
N3—H33	0.89 (1)	C13—C14	1.395 (5)
N4—C14	1.390 (4)	C13—H13	0.9500
N4—H41	0.88 (1)	C15—H15A	0.9800
N4—H42	0.87 (3)	C15—H15B	0.9800
N5—C17	1.464 (4)	C15—H15C	0.9800
N5—H51	0.88 (1)	C16—H16A	0.9800
N5—H52	0.89 (1)	C16—H16B	0.9800
N5—H53	0.89 (1)	C16—H16C	0.9800
N6—C22	1.388 (4)	C17—C18	1.379 (5)
N6—H61	0.88 (3)	C17—C22	1.395 (5)
N6—H62	0.88 (1)	C18—C19	1.392 (4)
C1—C6	1.387 (4)	C18—H18	0.9500
C1—C2	1.407 (5)	C19—C20	1.407 (5)
C2—C3	1.384 (4)	C19—C23	1.505 (5)
C3—C4	1.397 (4)	C20—C21	1.383 (5)
C3—H3	0.9500	C20—C24	1.511 (4)
C4—C5	1.396 (5)	C21—C22	1.398 (4)
C4—C7	1.505 (4)	C21—H21A	0.9500
C5—C6	1.391 (4)	C23—H23A	0.9800
C5—C8	1.510 (4)	C23—H23B	0.9800
C6—H6	0.9500	C23—H23C	0.9800
C7—H7A	0.9800	C24—H24A	0.9800
C7—H7B	0.9800	C24—H24B	0.9800
C7—H7C	0.9800	C24—H24C	0.9800
			
H1—O1—H2	107.4 (17)	C9—C10—C11	121.5 (3)
C1—N1—H11	113 (3)	C9—C10—H10	119.2
C1—N1—H12	121 (3)	C11—C10—H10	119.2
H11—N1—H12	110.0 (17)	C10—C11—C12	118.1 (3)
C2—N2—H21	118 (3)	C10—C11—C15	120.4 (3)
C2—N2—H22	114 (3)	C12—C11—C15	121.5 (3)
H21—N2—H22	111.0 (17)	C13—C12—C11	120.0 (3)
C9—N3—H31	110 (2)	C13—C12—C16	119.2 (3)
C9—N3—H32	113 (2)	C11—C12—C16	120.9 (3)
H31—N3—H32	107.1 (15)	C12—C13—C14	122.4 (3)
C9—N3—H33	111 (2)	C12—C13—H13	118.8
H31—N3—H33	108.4 (15)	C14—C13—H13	118.8
H32—N3—H33	107.4 (14)	N4—C14—C13	121.9 (3)
C14—N4—H41	120 (2)	N4—C14—C9	121.0 (3)
C14—N4—H42	115 (2)	C13—C14—C9	116.9 (3)
H41—N4—H42	111.9 (16)	C11—C15—H15A	109.5
C17—N5—H51	108 (2)	C11—C15—H15B	109.5
C17—N5—H52	110 (2)	H15A—C15—H15B	109.5
H51—N5—H52	108.5 (15)	C11—C15—H15C	109.5
C17—N5—H53	113 (2)	H15A—C15—H15C	109.5
H51—N5—H53	109.2 (15)	H15B—C15—H15C	109.5
H52—N5—H53	108.2 (14)	C12—C16—H16A	109.5
C22—N6—H61	119 (2)	C12—C16—H16B	109.5
C22—N6—H62	114 (2)	H16A—C16—H16B	109.5
H61—N6—H62	110.3 (16)	C12—C16—H16C	109.5
C6—C1—N1	121.6 (3)	H16A—C16—H16C	109.5
C6—C1—C2	118.9 (3)	H16B—C16—H16C	109.5
N1—C1—C2	119.4 (3)	C18—C17—C22	122.0 (3)
C3—C2—C1	118.7 (3)	C18—C17—N5	119.6 (3)
C3—C2—N2	121.2 (3)	C22—C17—N5	118.4 (3)
C1—C2—N2	119.8 (3)	C17—C18—C19	121.1 (3)
C2—C3—C4	122.3 (3)	C17—C18—H18	119.5
C2—C3—H3	118.9	C19—C18—H18	119.5
C4—C3—H3	118.9	C18—C19—C20	117.9 (3)
C3—C4—C5	119.0 (3)	C18—C19—C23	120.1 (3)
C3—C4—C7	119.9 (3)	C20—C19—C23	121.9 (3)
C5—C4—C7	121.2 (3)	C21—C20—C19	120.0 (3)
C6—C5—C4	118.7 (3)	C21—C20—C24	119.4 (3)
C6—C5—C8	120.3 (3)	C19—C20—C24	120.6 (3)
C4—C5—C8	120.9 (3)	C20—C21—C22	122.5 (3)
C1—C6—C5	122.5 (3)	C20—C21—H21A	118.8
C1—C6—H6	118.8	C22—C21—H21A	118.8
C5—C6—H6	118.8	N6—C22—C17	121.8 (3)
C4—C7—H7A	109.5	N6—C22—C21	121.6 (3)
C4—C7—H7B	109.5	C17—C22—C21	116.5 (3)
H7A—C7—H7B	109.5	C19—C23—H23A	109.5
C4—C7—H7C	109.5	C19—C23—H23B	109.5
H7A—C7—H7C	109.5	H23A—C23—H23B	109.5
H7B—C7—H7C	109.5	C19—C23—H23C	109.5
C5—C8—H8A	109.5	H23A—C23—H23C	109.5
C5—C8—H8B	109.5	H23B—C23—H23C	109.5
H8A—C8—H8B	109.5	C20—C24—H24A	109.5
C5—C8—H8C	109.5	C20—C24—H24B	109.5
H8A—C8—H8C	109.5	H24A—C24—H24B	109.5
H8B—C8—H8C	109.5	C20—C24—H24C	109.5
C10—C9—C14	121.1 (3)	H24A—C24—H24C	109.5
C10—C9—N3	121.1 (3)	H24B—C24—H24C	109.5
C14—C9—N3	117.8 (3)		
			
C6—C1—C2—C3	−0.9 (5)	C11—C12—C13—C14	0.5 (5)
N1—C1—C2—C3	174.7 (3)	C16—C12—C13—C14	−178.7 (3)
C6—C1—C2—N2	173.5 (3)	C12—C13—C14—N4	174.6 (3)
N1—C1—C2—N2	−10.9 (5)	C12—C13—C14—C9	0.0 (5)
C1—C2—C3—C4	1.0 (5)	C10—C9—C14—N4	−175.3 (3)
N2—C2—C3—C4	−173.4 (3)	N3—C9—C14—N4	1.9 (5)
C2—C3—C4—C5	−0.3 (5)	C10—C9—C14—C13	−0.6 (5)
C2—C3—C4—C7	179.7 (3)	N3—C9—C14—C13	176.6 (3)
C3—C4—C5—C6	−0.5 (5)	C22—C17—C18—C19	−1.1 (5)
C7—C4—C5—C6	179.5 (3)	N5—C17—C18—C19	−179.3 (3)
C3—C4—C5—C8	178.8 (3)	C17—C18—C19—C20	−1.0 (5)
C7—C4—C5—C8	−1.2 (5)	C17—C18—C19—C23	179.0 (3)
N1—C1—C6—C5	−175.3 (3)	C18—C19—C20—C21	2.0 (5)
C2—C1—C6—C5	0.1 (5)	C23—C19—C20—C21	−178.1 (3)
C4—C5—C6—C1	0.6 (5)	C18—C19—C20—C24	−176.8 (3)
C8—C5—C6—C1	−178.8 (3)	C23—C19—C20—C24	3.2 (5)
C14—C9—C10—C11	0.8 (5)	C19—C20—C21—C22	−0.8 (5)
N3—C9—C10—C11	−176.3 (3)	C24—C20—C21—C22	177.9 (3)
C9—C10—C11—C12	−0.3 (5)	C18—C17—C22—N6	−173.9 (3)
C9—C10—C11—C15	178.3 (3)	N5—C17—C22—N6	4.2 (5)
C10—C11—C12—C13	−0.3 (5)	C18—C17—C22—C21	2.3 (5)
C15—C11—C12—C13	−178.9 (3)	N5—C17—C22—C21	−179.6 (3)
C10—C11—C12—C16	178.8 (3)	C20—C21—C22—N6	174.9 (3)
C15—C11—C12—C16	0.3 (5)	C20—C21—C22—C17	−1.3 (5)

### Hydrogen-bond geometry (Å, °)

**Table:**

*D*—H···*A*	*D*—H	H···*A*	*D*···*A*	*D*—H···*A*
O1—H1···Cl1	0.85 (2)	2.37 (2)	3.212 (3)	172 (4)
O1—H2···Cl1^i^	0.85 (4)	2.82 (3)	3.402 (3)	128 (4)
O1—H2···Cl1^ii^	0.85 (4)	2.73 (4)	3.331 (2)	129 (3)
N1—H12···Cl1^i^	0.88 (3)	2.81 (3)	3.661 (3)	164 (3)
N2—H21···Cl1	0.87 (2)	2.61 (3)	3.361 (3)	145 (3)
N2—H22···N1	0.87 (4)	2.49 (4)	2.810 (4)	102 (3)
N2—H22···Cl1^ii^	0.87 (4)	2.79 (4)	3.599 (3)	155 (3)
N3—H31···N2	0.89 (2)	2.08 (2)	2.927 (4)	160 (2)
N3—H32···Cl1^i^	0.89 (3)	2.22 (3)	3.092 (3)	168 (2)
N3—H33···Cl2	0.89 (2)	2.35 (2)	3.216 (3)	167 (2)
N4—H41···Cl2^i^	0.87 (2)	2.38 (3)	3.233 (3)	165 (3)
N4—H42···Cl1^i^	0.87 (3)	2.63 (3)	3.434 (3)	155 (3)
N5—H51···O1	0.88 (2)	1.88 (2)	2.758 (3)	172 (3)
N5—H52···Cl2^iii^	0.89 (2)	2.35 (2)	3.242 (3)	176 (2)
N5—H53···Cl2	0.89 (2)	2.75 (3)	3.176 (3)	111 (2)
N5—H53···Cl2^iv^	0.89 (2)	2.73 (2)	3.540 (3)	153 (2)
N6—H61···Cl2^iv^	0.88 (3)	2.71 (3)	3.408 (3)	138 (2)
N6—H62···N1^ii^	0.88 (2)	2.45 (3)	3.277 (5)	156 (3)

Symmetry codes: (i) *x*, *y*+1, *z*; (ii) −*x*+1/2, *y*+1/2, −*z*+1/2; (iii) −*x*+3/2, *y*−1/2, −*z*+1/2; (iv) −*x*+3/2, *y*+1/2, −*z*+1/2.
